# Fungal‐Driven Airways Dis‐Immunity From Asthma to Allergic Bronchopulmonary Aspergillosis: Dissecting Similarities and Differences. An EAACI Task Force Report

**DOI:** 10.1111/all.16687

**Published:** 2025-08-01

**Authors:** Marco Caminati, Isabella Annesi‐Maesano, Wojciech Feleszko, Giuseppe Guida, Christer Janson, Shadi Hägg, Andrei Malinovschi, Luciana Tanno, Joana Vitte, Francesca Ambrosani, Matteo Maule

**Affiliations:** ^1^ Allergy Unit and Asthma Center Verona Integrated University Hospital Verona Italy; ^2^ Department of Medicine University of Verona Verona Italy; ^3^ Institute Desbrest of Epidemiology and Public Health (IDESP), UMR1318 University of Montpellier and INSERM Montpellier France; ^4^ Department of Pulmonology, Allergy and Thoracic Oncology University Hospital of Montpellier Montpellier France; ^5^ IHU Immun4Cure (INSERM University of Montpellier, CHUM) Montpellier France; ^6^ Department of Paediatric Pneumonology and Allergy Medical University of Warsaw Medical University Children's Hospital Warsaw Poland; ^7^ Rare Lung Disease and Respiratory Pathophysiology Unit, Severe Asthma San Luigi Gonzaga University Hospital Turin Italy; ^8^ Department of Medical Sciences: Respiratory, Allergy and Sleep Research Uppsala University Uppsala Sweden; ^9^ Allergy Unit Hôpital Arnaud de Villeneuve, University Hospital of Montpellier Montpellier France; ^10^ WHO Collaborating Centre on Scientific Classification Support Montpellier France; ^11^ Laboratory of Immunology University Hospital of Reims Reims France; ^12^ INSERM UMR‐S 1250 P3CELL University of Reims Reims France

**Keywords:** allergic bronchopulmonary aspergillosis, *Aspergillus*, biologics, fungal sensitization, severe asthma

## Abstract

Severe asthma and allergic bronchopulmonary aspergillosis (ABPA) do not rarely coexist and share several similarities in terms of pathobiological background, together with overlapping clinical manifestations, misleading the correct diagnosis. Within that scenario, severe asthma with fungal sensitization (SAFS) further complicates the correct pheno‐endotyping, which still needs to be recognized in the light of the greater burden and higher risk of irreversible damage related to ABPA and SAFS when compared to asthma alone. The identification of pathobiological drivers underlying different conditions remains challenging; in fact, available biomarkers, although accurate when related to each specific condition, do not always fully support a clear‐cut differential diagnosis. The opportunity for innovative targeted treatments, although needing further evidence, should further stimulate a precise endo‐phenotyping of severe asthma patients presenting some hallmarks of fungal‐related dis‐immunity to provide them the best standard of care for preventing disease evolution and achieving complete remission. This review provides a comparative outline of the recent advances in terms of pathophysiology, clinical manifestations, biomarkers, and management of asthma and ABPA, including a focus on SAFS, with the aim of updating the practical approach to those conditions and supporting their correct recognition.

**Abbreviations**
A1AT: α1‐antitrypsine
AATD: α1‐antitrypsine deficiency
ABPA: allergic bronchopulmonary aspergillosis
AFAD: allergic fungal airway disease
AJ: adherens junction
ASM: airway smooth muscle
BEC: blood eosinophil count
CF: cystic fibrosis
COPD: chronic obstructive pulmonary disease
ECM: extracellular matrix
HAM: high‐attenuation mucus
Ig: immunoglobulin
mAB: monoclonal antibody
MMP: matrix metalloproteinase
SAFS: severe asthma with fungal sensitization
TJ: tight junction

## 1 Introduction

Allergic bronchopulmonary aspergillosis (ABPA) is a multiple‐driven dis‐immune condition primarily caused by an impaired reaction to *Aspergillus fumigatus* (AF), a common airborne fungus [1]. Although definitely less frequent when compared to asthma, it might be associated with that condition, and its clinical manifestations, especially in the early disease stages, might almost completely overlap, misleading the correct diagnosis [2, 3]. Which apparently does not represent a big mistake in light of the pathobiological similarities shared by asthma and ABPA, thus responding to similar treatment options [2]. Nevertheless, coexisting asthma‐ABPA evolution toward irreversible damage is more rapid and more relevant in terms of overall burden when compared to asthma alone, especially if not recognized, and requires a tailored treatment [3]. The typical difficult‐to‐treat asthma associated with an underlying ABPA is characterized by recurring major exacerbations leading to the frequent use of oral steroids, which may mask the hallmark of ABPA and prevent its timely recognition. The spectrum of airways conditions characterized by fungal sensitization (Allergic Fungal Airway Disease—AFAD) is, however, much broader, also including severe asthma with fungal sensitization (SAFS), a different condition from ABPA that further complicates the correct pheno‐endotyping, but still needing to be recognized as an asthma subtype showing a higher exacerbation rate and greater risk of lung function impairment when compared to severe eosinophilic asthma [4, 5].

In this review the authors, from the European Academy of Allergy and Clinical Immunology Task Force on diagnosis and therapeutic management of ABPA, will provide a comparative outline of the recent advances in terms of pathophysiology, clinical manifestations, biomarkers, and management of asthma and ABPA, including a focus on SAFS, with the aim of updating the practical approach of those conditions and supporting their correct recognition.

### 1.1 Sizing the Issue—Epidemiological Perspective

The diagnosis of ABPA is often challenging due to its overlapping clinical features with severe asthma and other pulmonary conditions, such as chronic obstructive pulmonary disease (COPD) and bronchiectasis. This overlap can result in misdiagnosis or delayed recognition, contributing to underdiagnosis and, consequently, an underestimated prevalence of the condition. Globally, the prevalence of ABPA is estimated to affect approximately 4.8 million people. This estimate primarily reflects cases among individuals with asthma (262 million asthmatic worldwide) and cystic fibrosis (CF) (70,000 to 100,000 people living with CF worldwide), where the condition is most frequently diagnosed [5].

Among asthma patients, a specific subset develops a hypersensitivity reaction to AF, a common environmental fungus, leading to ABPA, with a global prevalence of ABPA among asthma patients estimated to range between 1% and 3%, though rates can rise significantly in patients with severe or poorly controlled asthma, reaching up to 13% [2, 6]. In CF patients, prevalence rates are higher, typically ranging from 5% to 15%, depending on geographic location and screening practices [7].

ABPA can occasionally occur without coexisting asthma, particularly in individuals with other lung conditions or immunosuppression, though these cases are less common [5]. ABPA contributes to significant morbidity in affected individuals by accelerating the decline of lung function, increasing hospitalization rates, and leading to irreversible bronchiectasis if untreated [2, 5, 6, 8].

### 1.2 Risk Factors and Predisposing Conditions to ABPA Development

Several risk factors predispose individuals with asthma to develop ABPA. The most significant risk factor is sensitization to AF, which can be detected through skin testing or elevated specific IgE levels [2]. Sensitization is more common in individuals with severe, corticosteroid‐dependent asthma or those with frequent exacerbations. Other factors include genetic predisposition, structural lung abnormalities, environmental exposure, and immunological dysregulation (Table 1).

**TABLE 1:** Risk factors predisposing individuals with asthma to develop allergic bronchopulmonary aspergillosis (ABPA).

Risk factors	Observations	References
Genetic predisposition	Variants in genes related to immune regulation, such as *CFTR*, *IL‐4 receptor*, and *TLR polymorphisms*, have been associated with an increased risk of ABPA development	[9, 10]
Structural lung abnormalities	Pre‐existing conditions like bronchiectasis or chronic obstructive pulmonary disease (COPD) can increase susceptibility by impairing mucociliary clearance	[5, 11]
Environmental exposure	High levels of environmental *Aspergillus* spores, often found in humid, agricultural, or construction‐heavy environments, can increase colonization risk in susceptible individuals	[12, 13, 14]
Immunological dysregulation	ABPA development is associated with exaggerated T‐helper 2 (Th2) immune responses, leading to increased production of interleukins (IL‐4, IL‐5, IL‐13) and subsequent eosinophilic inflammation	[15]

### 1.3 Disease Evolution and Progression

From an epidemiological perspective, the disease evolution and progression of ABPA are influenced by several factors, including underlying respiratory conditions, environmental exposure, and early detection. In the early stages, ABPA symptoms often mimic poorly controlled asthma or early signs of lung disease in CF, leading to frequent underdiagnosis. From the environmental point of view, exposure to fungal spores and airborne spore distribution are expected to be exacerbated due to rising temperatures and changing humidity patterns related to climate change. In addition, higher population density and pollution levels in towns, also related to climate change, can contribute to increased fungal exposure and respiratory vulnerability.

## 2 Pathobiological Connections Between Asthma and ABPA in the Light of the Epithelial Barrier Dysfunction Concept

The airway epithelium is a key upstream regulator of type 2 inflammation. In response to stress and injury, several cytokines and alarmins are released that promote type 2 inflammation, including thymic stromal lymphopoietin (TSLP), IL (interleukin)‐25, and, notably, IL‐33 [16]. This process is common both in type 2 high asthma and ABPA and provides a potential treatment target in both asthma and ABPA.

Allergic asthma is universally associated with a polarized Th2 response characterized by an influx of eosinophils into the airway mucosa, mucus overproduction, and bronchial hyperresponsiveness (AHR) induced by Th2 cytokines such as IL‐4, IL‐5, IL‐13, and IL‐9. However, the recognition of eosinophilic‐driven non‐allergic (intrinsic) forms of asthma, with a later onset, a more severe clinical course in adults, a frequent association with nasal polyps, and aspirin idiosyncrasy, led to the demonstration of an innate Th2 cell‐independent response triggered by exposure to microbes, air pollution, cigarette smoke, chemicals, and diesel exhaust particles [17]. Actually, airway epithelial cells (AEC) constitute the first line of defense in the respiratory tract against various infectious pathogens, allergens, and physical insults. In recent years, it has emerged that the airway epithelium not only functions as a physical and passive barrier but also actively participates in the modulation of innate and adaptive immune responses [18]. AECs can recognize exogenous and endogenous danger signals PAMPs and DAMPSs (Pathogen‐associated and Damage‐associated molecular pattern, respectively) through specific receptors called PRRs (pattern recognition receptors). The asthmatic airway epithelium, once activated, releases Type‐2 alarmins that are able to recruit and activate different immune cells. The activation of type 2 innate lymphoid cells (ILC2s), mainly localized at the interface sites with the external environment, such as the bronchial submucosa, is crucial in eliciting Th2 cytokine production such as IL‐4, IL‐13, IL‐5, and stimulating IgE production by B lymphocytes independently of T helper lymphocytes (polyclonal IgE).

Exposure to β‐glucans, carbohydrates essential constituents of the fungal wall, appears to be by themselves a risk factor for asthma. β‐glucans act through a receptor, Dectin‐1, present on macrophages, neutrophils, and dendritic cells, which mediate phagocytosis signals, oxidative stress, and the synthesis of inflammatory cytokines such as IL‐6, IL‐8, IL‐12, IL‐18, and TNF‐α, and the activation of Th17 type lymphocytes. Therefore, β‐glucans drive innate immune cells to phagocytose and kill conidia as well as elicit a proinflammatory response [19]. Other *Aspergillus* cell wall components are able to engage different surface‐bound, endosomal, or cytosolic PRRs leading to activation of complement, opsonization, neutrophil recruitment, and inflammasome signals. However, in asthma with fungal sensitization, this anti‐fungal host defense program is counterbalanced by the TH2‐associated chemokine CCL17/TARC pathway, which not only recruits TH2 cells into the airways but also impairs macrophage killing of Aspergillus and favors its survival by Treg immunomodulation. A suggestive hypothesis is that in ABPA, high levels of CCL17 may aid in dampening the Th2 response and allow the triggering of Th1 and Th17 responses [20].

In addition to AEC's innate immune activation, epithelium dysfunction has been shown to be crucial in both asthma and ABPA pathogenesis. In asthma, the barrier functions can be compromised by partial disruption of tight junctions (TJs) by respiratory viruses and proteolytically active allergens, increasing epithelial permeability. The damage is enhanced by defective antioxidant pathways and aberrant repair activity of basal cells, leading to chronic inflammation. Allergens with an inherent protease activity disrupt the epithelial layer and penetrate the airway mucosa, favoring the sensitization mechanism [17].

*Aspergillus* contains elastinolytic serine proteases with elastase activities (Asp f 13) able to cleave epithelial tight (TJ) and adherens (AJs) junctions and to produce mitochondrial reactive oxygen species, leading to epithelial damage. In the airways of predisposed individuals, the activation of protease‐activated receptors (PARs) by fungal serine proteases leads to the release of alarmins, impairment of mucociliary clearance, Th2 polarized signals, and calcium induced expressions of pro‐inflammatory cytokines. In addition, neutrophil activation is critical in driving *Aspergillus* phagocytosis, oxidative stress response, and cytokine release by Dectin‐β‐glucan recognition. It has been shown that serine proteases from AF may cleave and inactivate the hDectin‐1 isoform, causing impairment of the complement system to clear AF from the lung, thus representing a predisposing condition to airways colonization or AF‐related disease exacerbations [21]. Second, the protease activity of AF in airway epithelial cells may lead to increased expression of MUC5AC, enhanced by the reduction of serine protease inhibitors. Allergen serine proteases (Asp f 13) from AF can also proteolytically disrupt airway smooth muscle (ASM) and extracellular matrix (ECM) by degrading collagen I and fibronectin or by activating endogenous matrix metalloproteinases‐9 (MMPs‐9) contributing to structural changes and remodeling of the asthmatic airway by degradation of ECM components [22].

Allergic airways are subjected chronically to the effects of proteases released by immune cells, such as elastase secreted from neutrophils. The protective effect of endogenous protease inhibitors that neutralize aberrant proteolytic activities is impaired by multiple mechanisms, leading to an anti‐protease and protease imbalance increasing the risk of irreversible lung tissue damage [23].

The imbalance of protease–antiprotease activities amplifies the process through the reduced expression of α1‐antitrypsin (A1AT) and serine protease inhibitor Kazal‐type 5 (SPINK5). Interestingly, some experimental data showed the ability of Aspergillus niger strains to produce human A1 Proteinase Inhibitor in a kind of autocrine regulation [24]. A1AT deficiency (AATD) has been associated with severe forms of asthma [25] and, in addition, both AATD and ABPA represent a diagnostic challenge of bronchiectasis [26]; yet the clinical correlation between endogenous protease inhibitors downregulation and ABPA has not been systematically described, and the mutual relationship is unknown or unreported in recent extensive cohorts of ABPA patients [3, 27]. Therefore, the clinical role of endogenous protease imbalance and potential strategies to rectify protease inhibitors deficiency represent an unmet need in ABPA management.

Sub‐chronic inhalatory exposure to *A. fumigatus* has been shown to stimulate bronchial AECs by the activation of protease receptor (PAR‐2) to abundantly produced IL‐33, an alarmin with a recognized role in causing immune‐related tissue damage in the respiratory system. IL‐33 is also able to recruit ILC2 to the airway, stimulating IL‐13 and IL‐5 production. In asthma, TSLP is a key alarmin which enhances Type‐2 immunity progression of allergic disease and alterations resulting in airway remodeling. One study was able to show that AF spores stimulate increased secretion of TSLP compared to Alternaria, therefore suggesting differential AECs activation in fungal asthma compared to ABPA [28].

Another interesting point regarding ABPA pathogenesis is the exaggerated IgG response against fungal antigens that reflects persistent AF airway colonization. Positive AF IgG or precipitating antibodies are included in ABPA diagnostic criteria, as a surrogate of type III hypersensitivity and the formation of immune complexes. Specific AF IgG4 and IgA, as well as IgG directed to ribotoxin Asp f 1, have also been detected in ABPA patients compared to AF sensitized asthmatics without ABPA and further contribute to ABPA pathobiological burden [29].

## 3 Clinical Hallmarks, Biomarkers and Molecular Diagnosis

**FIGURE 1:**
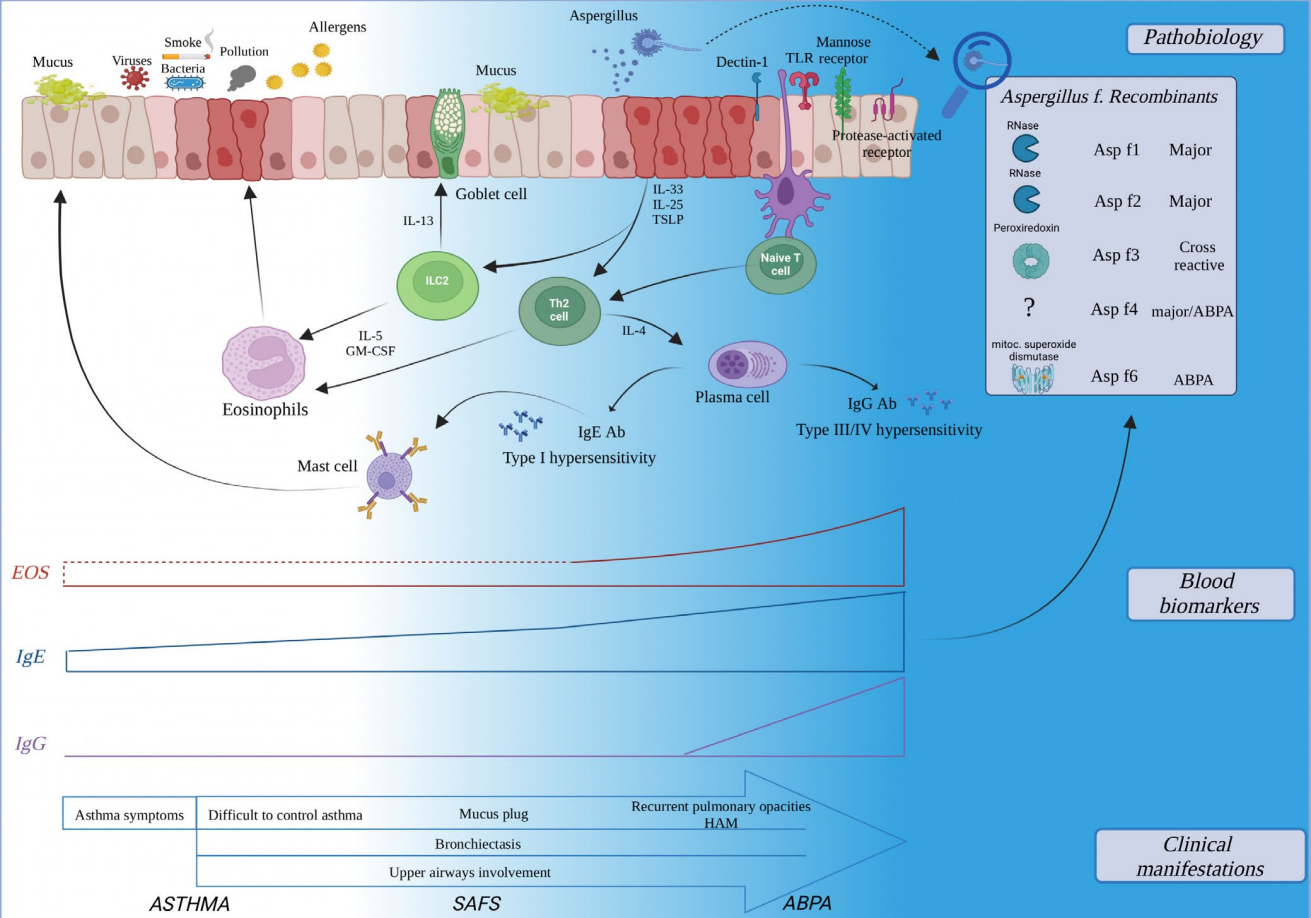
Synoptical overview of pathobiological mechanisms, biomarkers, and clinical manifestation across asthma and fungal‐driven airways dis‐immunity. ABPA, bronchopulmonary allergic aspergillosis; Eos, blood eosinophils; HAM, high‐attenuation mucus; SAFS, severe asthma with fungal sensitization.

The natural history of ABPA is that of a remitting–relapsing disease, reflecting the complex, multiple‐driven immunological background described above (Figure 1). Combining clinical manifestations with immunological and radiological findings is essential to define ABPA diagnosis and ensure its proper follow‐up.

On a clinical ground, frequent manifestations in patients with ABPA are wheeze, cough, and expectoration; these symptoms are also common in asthma, but when occurring with a recurrent trajectory, ABPA should be assessed or re‐assessed. Some symptoms that are more specific for ABPA include hemoptysis and expectoration of brownish mucus plugs. Hemoptysis may occur in 8% to 50% of the ABPA patients, while brown mucus plugs occur in 28% to approximately two‐thirds of the patients [30, 31] (Figure 1). Clinical suspicion of ABPA is further supported by well‐identified predisposing conditions besides asthma, including CF, COPD, and bronchiectasis [2, 3, 32]. Of note, ABPA was found in 6% of European patients with bronchiectasis and AF sensitization [33], and current guidelines from the European Respiratory Society recommend testing for ABPA in all newly diagnosed cases of adult bronchiectasis [34].

ABPA natural history consists of flare and remission episodes leading to irreversible lung damage with central bronchiectasis and fibrosis if not appropriately treated. In fact, common computed tomography findings that have been highlighted in ABPA are central bronchiectasis, mucus plugs, or high mucus attenuation [35]. However, a similar clinical presentation has been found in ABPA patients, with and without bronchiectasis [36], which suggests a room for early identification and prevention of irreversible lung impairment. The relevance of detecting radiological abnormalities is further supported by the evidence that mucus plugs in association with poorer lung function and longer duration of asthma were related to increased likelihood of exacerbations [30]. Similarly, in a study of ABPA patients with central bronchiectasis already developed, individuals with HAM presented higher BEC, higher rates of AF detection isolated in sputum, and expectoration of brownish‐black mucus plugs, more affected lobes and segments, poorer pulmonary function, and a higher rate of relapse than the group with low attenuation mucus [37]. Atelectasis and lung collapse can be found in ABPA [38, 39]. Of note, many of the radiological abnormalities detected in ABPA, such as bronchial wall thickening, bronchiectasis, mucus plugging, mosaic perfusion, and expiratory air trapping, are as well part of the clinical spectrum of primary antibody deficiency. In that case, defective IgG response represents a predisposing condition for pulmonary diseases mediated by AF as an infectious pathogen. Secondary colonization or infections with *Aspergillus* species may lead to chronic airway infection and (ABPA)‐like pattern in these patients [40]. This is the case of Pulmonary Aspergillosis complicating Hyper‐IgE Syndrome and Autosomal Dominant STAT3 Deficiency [32]. However, immune deficiency is not a predisposing condition for ABPA, a disease that is not an infection, is not invasive, and does not spread systemically.

ABPA biomarkers reflect the overall immune response polarized towards type 2 inflammation, with excess type 2 cytokines, mainly IL‐4, and insufficient T regulatory and type 3 (Th17) responses [41, 42].

Fractional exhaled nitric oxide is a marker of type 2 airways inflammation, and elevated FeNO levels are produced by airway epithelial cells inducible nitric oxide synthetase in response to high IL‐4 and/or IL‐13 [43]. Patients with type 2 asthma have elevated FeNO levels, and FeNO is useful as a diagnostic aid [44] or for guiding therapeutic decisions [45]. Patients with ABPA without asthma also have elevated FeNO levels, and in two Chinese studies, they appear to have even higher levels than patients with asthma sensitized to AF [29, 46]. Moreover, higher levels of FeNO (> 57 parts per billion) appear to be related to the need for longer durations of oral corticosteroid treatment and a higher likelihood of relapse/exacerbations [47]. When considering the antibody‐mediated response, multiple biomarkers are needed to assess the complex pathophysiology of ABPA, with type I (IgE‐mediated) and type III (IgG‐mediated) adaptive immune responses to AF in a context of type 2 inflammation. The original criteria for diagnosing ABPA proposed by Rosenberg Paterson [48], and the ISHAM‐ABPA criteria, which are nowadays widely used [8] basically integrate clinical hallmarks and biomarkers. Other versions of these criteria have been proposed [49] as well as criteria from the Japanese ABPM group [35]; however, maintaining a similar combined approach.

ABPA is an allergic fungal disease characteristically associating high AF‐specific IgE and IgG responses in the context of a significant elevation of total IgE and usually occurring against the backdrop of a predisposing pulmonary condition.

**FIGURE 2:**
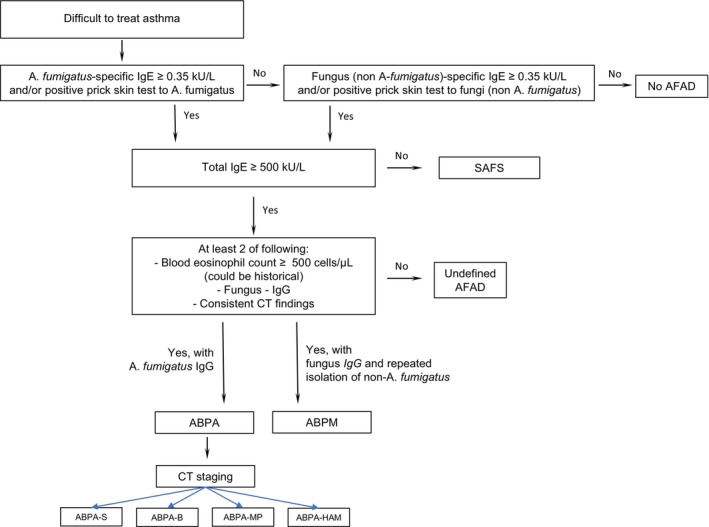
Diagnostic procedure for diagnosing Allergic Bronchopulmonary Aspergillosis. ABPA, Allergic Bronchopulmonary Aspergillosis; ABPA‐B, Allergic Bronchopulmonary Aspergillosis with bronchiectasis; ABPA‐HAM, Allergic Bronchopulmonary Aspergillosis with high‐attenuation mucus; ABPA‐MP, Allergic Bronchopulmonary Aspergillosis with mucus plugging; ABPA‐S, Serologic Allergic Bronchopulmonary Aspergillosis; ABPM, Allergic Bronchopulmonary Mycosis; AFAD, Allergic Fungal Airway Disease; CT, computed tomography; SAFS, severe asthma with fungal sensitization.

It is currently understood that effective screening for ABPA starts with the assessment of AF‐specific IgE, and if the serum AF IgE is greater than 0.35 kUA/L, total IgE greater than 500 kIU/L is checked at the next step (Figure 2) [8, 50]. Alternatively, total IgE may be assessed first, followed by AF‐specific IgE [51]. Levels of AF‐specific IgE greater than 20 kUA/L at ABPA diagnosis in treatment‐naïve patients were found to predict failure to achieve remission at 6 months [52]. Of note, especially in the presence of a confirmed asthma diagnosis, high IgE and BEC, positive AF‐specific IgE, support the early identification of ABPA even before the occurrence of radiological abnormalities and irreversible damage (ABPA‐S), thus allowing to potentially prevent disease progression [51, 53, 54].

Humans inhale up to thousands of *A. fumigatus* spores daily, but promptly reject most of them into exhaled air, unless airway conditions favor their local persistence (epithelial lesions, increased secretion and/or decreased fluidity of mucus, ongoing local inflammation) [1]. AF is a “thermophilic” fungus, that is, it proliferates at 37°C as opposed to *Alternaria alternata* or *Cladosporium herbarum*, which exhibit lower optimal temperatures.

The demonstration of AF in the airways through culture, PCR, or fungal hyphae visualization has been repeatedly associated with ABPA, and antifungals have long been used as efficient therapy in addition to corticosteroids, which constitute the first‐line treatment [8]. Live AF spores at early stages of germination activate airway dendritic cells to induce allergic inflammation [55]. The induction of allergen‐specific IgE and IgG able to bind AF as an intact organism, or as immunologically active fractions containing allergenic molecules, results in hypersensitivity symptoms and persistent type 2 inflammation.

The demonstration of non‐invasive fungal hyphae in mucus plugs was proposed as a biomarker for the diagnosis of ABPA, also effective for allergic broncho‐pulmonary mycosis (ABPM) due to non‐*Aspergillus* fungi [35]. The modified diagnostic criteria for ABPA/ABPM comprising the demonstration of hyphae exhibited improved diagnostic sensitivity and specificity compared with Rosenberg‐Patterson's and the 2013 update [56]. Importantly, the absence of fungal hyphae in mucus also helped distinguish between ABPA and SAFS in cases when fungal growth was present in sputum or broncho‐alveolar lavage from the latter (5/15 cases in the study).

Thus, the effect of antifungals in ABPA is best understood as an “exposomic intervention”, decreasing the burden of live AF and thus the burden of immunogenic live fungi and allergenic components released in the airways.

**FIGURE 3:**
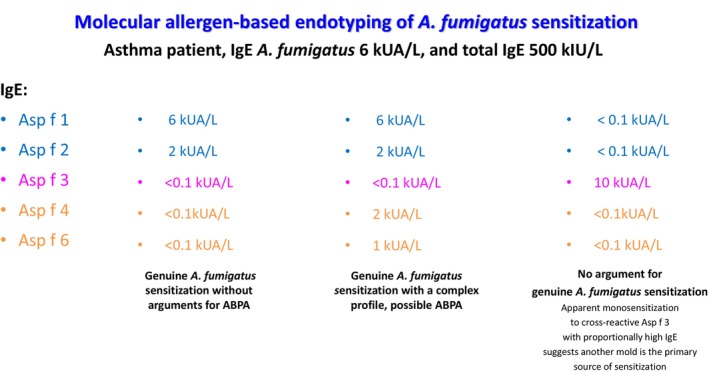
Clinical vignette summarizing the potential differential diagnosis scenarios supported by the molecular sensitization profile.

The endotype of AF sensitization can be investigated using molecular allergens (components). In addition to their pathophysiological role, AF components provide in vitro diagnostic tools to improve the specificity of the ABPA screening and diagnosis [51, 53, 54]. Five AF components (molecular allergens) are commercially available [57]. Two of them, the mitogillin (ribotoxin) Asp f 1 and Asp f 2 of unknown biological function, are markers of genuine sensitization to AF, meaning that the demonstration of Asp f 1 and/or Asp f 2‐specific IgE, with reasonable concordance to extract‐specific IgE, confirms primary sensitization to AF. Asp f 1 and Asp f 2 are major AF allergens, meaning that virtually all subjects genuinely sensitized to AF display detectable IgE to at least one of these two allergens. A third one, Asp f 4, is also a marker of genuine AF sensitization, but IgE to Asp f 4 is specific to ABPA. In other words, AF‐sensitized patients display IgE to Asp f 1 and Asp f 2 even if they are not affected by ABPA, but the detection of Asp f 4 IgE in addition to Asp f 1 and/or Asp f 2 supports the diagnosis of ABPA [32, 58, 59]. Asp f 3 is a peroxysomal protein with high cross‐reactivity among multiple fungal species. Detection of Asp f 3 IgE in a subject with AF sensitization strongly suggests a distinct fungal species is the primary sensitizer if concomitant Asp f 1 and/or Asp f 2 IgE are not detected; if they are, it is interpreted as a more complex molecular profile, usually associated with more pronounced molecular spreading, long‐standing AF sensitization, and higher IgE levels. Conversely, apparent monosensitization to Asp f 3 IgE is a strong indicator of a primary fungal species sensitizer distinct from AF. In the clinical setting, testing for *Alternaria alternata* (marker allergen Alt a 1) helps identify the culprit primary sensitizer in most cases. Finally, Asp f 6 displays some cross‐reactivity with other members of the Mn superoxide family but has been convincingly associated with ABPA when Asp f 1 and/or Asp f 2 IgE are demonstrated [60]. Figure 3 provides a clinical vignette summarizing the potential differential diagnosis scenarios supported by the molecular sensitization profile.

AF molecular allergens are part of the current standards for the investigation of *A. fumigatus* sensitization for ABPA screening, diagnosis, and follow‐up [51].

Functional investigations using basophil activation tests with whole AF extracts or AF molecular allergens mainly Asp f 4 to identify ABPA have reported good performance in most cohorts [61, 62, 63, 64].

### 3.1 Eosinophils: Relevance in Phenotyping, Disease Assessment and Potential Trajectory

Blood eosinophil count (BEC) plays a major role in asthma overall management, including phenotype identification and follow‐up over time [65]. Although not fully reflecting the complexity of asthma's immunological background and the underlying drivers of inflammation, which partially hampers their accuracy, eosinophils are currently considered the most relevant and easily available asthma biomarker [66, 67].

With or without the coexistence of elevated IgEs and FeNO levels, increased BEC connotates a T2 phenotype which characterizes the majority of asthma patients, particularly considering severe asthma. In the context of T2 asthma, elevated eosinophil levels are associated with a higher risk of asthma exacerbations and disease progression [68]. In terms of treatment selection, eosinophilic asthma is, generally speaking, more sensitive to steroid treatment compared to non‐T2 phenotypes and predicts or associates with a good clinical response to monoclonal antibodies targeting T2 cytokines [69].

In ABPA, the pathobiological relevance of eosinophils is even more relevant. The eosinophilic inflammatory component appears to be consistent in ABPA, disregarding whether ABPA is sans asthma or coexisting with non‐atopic or atopic asthma, according to a recent Japanese study [70]. An increased BEC is almost invariably present and represents a diagnostic criterion [8]. With respect to severe eosinophilic asthma, ABPA patients often show a greater eosinophilic burden, and the presence of bronchiectasis and high‐attenuation mucus (HAM) is associated with higher BEC, typically greater than 1 × 10^9^/L [71].

In addition, intense eosinophilic infiltrates in pathological lung samples of ABPA patients [72] are commonly described. In fact, eosinophils are recruited in the airways by the inflammatory cascade triggered by the fungus exposure (Figure 1).

Through ETosis, a programmed cell death defense mechanism characterized by chromatin expulsion, eosinophils are thought to be the major cause of the dense mucus that characterizes ABPA patients, as their'trap' are thicker and less susceptible to digestion than the neutrophils' counterpart [73, 74]. Indirect evidence of this is provided by the significant improvement of mucus plugging in patients treated with anti‐eosinophil monoclonal antibodies (mAbs) [75], thus suggesting the presence of mucus plugging and HAM are clinical characteristics of marked eosinophilic inflammation.

In terms of biomarkers, blood hypereosinophilia alone is not sufficient to differentiate asthma and ABPA, although usually more marked in the latter, and should be contextualized within a more extensive evaluation including clinical‐radiological features and other biomarkers such as total IgE and AF specific IgE (Table 2; Figures 2 and 3). A recently published study explored potential biomarkers cut offs able to distinguish asthma from ABPA: no one of the biomarkers alone, including BEC, total IgEs, and specific IgEs demonstrated to be accurate enough, but a combination of the three yielded a specificity of 100%, with cut offs being 2347 IU/mL for total IgE, 1.91 kUA/L for AF specific IgE, and 507 cells/μL for BEC [76].

**TABLE 2:** Distinguishing severe asthma with fungal sensitisation (SAFS) from allergic bronchopulmonary aspergillosis (ABPA).

	ASTHMA	SAFS	ABPA
Specific IgE	≥ 0.35 kU/L in allergic/atopic	≥ 0.35 kU/L against any fungal species	≥ 0.35 kU/L against aspergillus
Total IgE	High in allergic/atopic	≤ 500 kU/L	> 500 kU/L
IgG	Negative	Negative against aspergillus	Positive against aspergillus
Eos	Variable	≤ 0.5 × 10^9^/L	Usually > 0.5 × 10^9^/L
Thoracic CT	Normal	Normal	Bronchiectasis, mucus plug, HAM, fleeting opacities
Asthma severity	Mild, moderate or severe	Severe	Mild, moderate or severe

Abbreviations: CT, computer tomography; Eos, blood eosinophils; HAM, high attenuation mucus.

However, on a practical ground, an increased BEC detected during the asthma follow‐up, especially when associated with the occurrence of poor or loss of disease control, should be carefully investigated as a potential marker of ABPA onset or exacerbation by exploring further ABPA hallmarks. Of note, every “common” asthma exacerbation is usually characterized by increased BEC levels [68] but it should not be neglected that the same scenario might suggest a potential trajectory of asthma evolution towards other eosinophilic conditions involving the lung, including ABPA, eosinophilic pneumonia, or eosinophilic granulomatosis with polyangiitis [65, 77, 78]. On the other side, especially when considering severe asthma patients, BEC might be biased by the ongoing background therapy, whether based on systemic steroids or monoclonal antibodies, showing poor correlation with the overall disease control. In fact, according to the last update of ABPA diagnostic criteria, eosinophils are listed among the “other components”, meaning that in the case of BEC < 0.5 × 10^9^/L, it does not necessarily exclude ABPA [8]. Thus, in the case of recurrently relapsing asthma, even in the absence of substantial BEC fluctuation, especially in the presence of suggestive clinical manifestations, radiological evaluation, as well as specific IgE molecular profiling should be performed in order to exclude ABPA.

### 3.2 SAFS and ABPA: Two Sides of the Same Coin?

Several allergic fungal conditions may affect the airways and are identified under the Allergic Fungal Airway Disease (AFAD) umbrella (Figure 2). The definition encompasses quite a broad spectrum of different diseases sharing the development of specific IgE against *Aspergillus* or Aspergillus‐like fungi but expressing different degrees of pathobiological impairment and symptom severity, from sensitization without other signs of airway disease to bronchial and lung involvement [79]. When managing asthma patients, ABPA and SAFS represent the two major conditions to be potentially unmasked due to their impact on asthma burden and evolution.

ABPA is the most known and most well studied, but other similar conditions, driven by fungi other than AF, may complicate asthma [79].

Most similar to ABPA is allergic bronchopulmonary mycosis (ABPM), which is an ABPA‐like syndrome that involves sensitization to fungus other than to AF [8]. Fungal sensitization is associated with having more severe asthma [80], and the term severe asthma with fungal sensitization (SAFS) has been developed for patients that have severe asthma in combination with IgE sensitization to fungal allergens but that do not fulfill the criteria for ABPA or ABPM [6] (Figure 2).

In the European Community Respiratory Health Survey conducted between 1990 and 1994, the median prevalence of skin prick test assessed sensitization to *Alternaria alternata* and *Cladosporium herbarum* was 3.3% and 1.7%, respectively [81], while the prevalence in the Global Allergy and Asthma European Network study conducted more than 10 years later was 6.1% in those < 45 years and 1.6% in those 45 years or older [82]. The other dominating fungal species associated with fungal allergy are *Aspergillus*, *Candida*, and *Penicillium* [83]. The prevalence of fungal sensitization in people with asthma is at least three times higher than in the general population. In a meta‐analysis in 2023, Agarwal and colleagues reported a prevalence of *Aspergillus* sensitization of 25% in people with asthma [84]. ABPA was found in 37% of asthmatics, with *Aspergillus* sensitization [84].

The diagnostic procedure for distinguishing SAFS from ABPA, within the spectrum of AFAD, includes an evaluation of asthma severity, blood sampling for assessing specific and total IgE, fungal specific IgG, peripheral blood eosinophilia (B‐Eos) and thoracic computer tomography (Figure 2). The distinction between SAFS and ABPA is not always clear, but in a review from 2011, Hogan and Devan suggested that total immunoglobulin E (IgE) is usually > 1000 kU/L in ABPA and < 1000 kU/L in SAFS [85]. In more recent guidelines, the threshold of total IgE for distinguishing ABPA from SAFS was set at 500 kU/L [8]. Another component of ABPA is its radiological presentation (bronchiectasis, mucus plugging, fleeting opacities), which are characteristics seldom present in SAFS [6]. Fungal‐specific IgG and B‐Eos > 0.5 × 10^9^/L support the ABPA diagnosis [8], while B‐Eos in SAFS, although higher than in non‐sensitized patients with asthma, is often < 0.5 × 10^9^/L [86]. Another difference between ABPA and SAFS is that ABPA can occur in patients with mild and moderate asthma, while patients with SAFS, by definition, always have severe asthma [87]. These differences between ABPA and SAFS are summarized in Table 2.

ABPA patients can be treated with only standard asthma therapy in a stable phase. The recommended treatment of SAFS is the same as other kinds of severe asthma, including treatment with inhaled corticosteroids, long‐acting beta‐2 agonists, anti‐leukotrienes, and long‐acting muscarinic antagonists, and, if needed, oral corticosteroids and biological treatment with anti‐IL5 drugs or omalizumab should be considered [6].

In the acute phase, ABPA is treated with oral corticosteroids or antifungal drugs, usually itraconazole. In contrast to ABPA, there is not extensive evidence supporting the use of antifungal treatment in SAFS. The studies showing improvement have been small, and the effect is limited [6].

Although similar in some ways, SAFS and ABPA are distinct clinical presentations of allergic fungal disease, and care should be taken to differentiate between the two.

## 4 Pediatric Perspective

While ABPA is well characterized in adults, its pediatric presentation remains underrecognized, leading to delays in diagnosis and treatment. Children with ABPA often exhibit persistent, poorly controlled asthma with frequent exacerbations, which can mimic other respiratory conditions. Consequently, understanding when to suspect ABPA in asthmatic children and whether diagnostic criteria should differ from adults is crucial for early intervention and improved outcomes.

### 4.1 Epidemiology and Risk Factors

The prevalence of ABPA in pediatric asthma is difficult to estimate due to underdiagnosis. Studies suggest that ABPA occurs in approximately 1%–5% of children with asthma and in 2%–15% of those with CF [88, 89]. Pediatric ABPA often manifests in school‐aged children and adolescents, with a mean age of diagnosis around 12 years [88]. Key risk factors for ABPA in children include:

- Asthma severity: Children with frequent exacerbations and high corticosteroid use are at greater risk.
- CF and airway colonization: *Aspergillus* colonization, particularly in CF patients, significantly increases ABPA risk [7].
- Environmental exposures: High mold exposure and indoor dampness have been linked to increased ABPA prevalence [88].
- Genetic predisposition: Specific human leukocyte antigen (HLA) alleles may confer susceptibility, although data in children remain limited [90].

### 4.2 Clinical Presentation and ABPA Hallmarks in Asthmatic Children

Pediatric ABPA often masquerades as steroid‐dependent or therapy‐resistant asthma, leading to misdiagnosis. Early detection is critical, as untreated pediatric ABPA can lead to progressive lung damage and bronchiectasis [91].

The following red flags should prompt suspicion of ABPA in asthmatic children:

1. Poorly controlled asthma despite appropriate inhaled therapy [88]
2. Recurrent pulmonary infiltrates on imaging, often mistaken for pneumonia
3. Persistent or recurrent cough with mucus plugging
4. Wheezing with systemic symptoms such as low‐grade fever, fatigue, or weight loss [56]
5. Peripheral eosinophilia and elevated total IgE levels that are disproportionate to the asthma severity
6. History of hemoptysis, particularly in adolescents [92]

### 4.3 Diagnostic Considerations: Should Pediatric Criteria Differ From Adults?

The diagnosis of ABPA in children largely follows adult criteria, but some adaptations may improve their sensitivity:

- IgE thresholds: a lower total IgE cutoff may be more appropriate for children, given their evolving immune responses [88].
- Exhaled nitric oxide (FeNO): Emerging evidence suggests that FeNO may help differentiate ABPA from asthma in children, though further validation is needed [93].
- Radiological findings: Chest CT remains essential, with central bronchiectasis as a hallmark, but transient infiltrates are more common in children [89].
- Molecular biomarkers: Advances in molecular diagnostics, including *Aspergillus*‐specific IgE and PCR‐based fungal assays, may improve early detection in children [51]

### 4.4 Future Directions

Pediatric ABPA remains underdiagnosed, necessitating heightened clinical awareness and refinement of pediatric‐specific diagnostic criteria. Advances in non‐invasive biomarkers, biologic therapies, and personalized medicine hold promise for optimizing treatment. Large‐scale pediatric studies are needed to establish evidence‐based management protocols distinct from adult guidelines [94].

## 5 Targeted Therapies

Similarly to asthma management, inflammatory response suppression does represent a major goal in ABPA treatment and can be achieved by the use of glucocorticoids and/or anti T2 biologic agents. In addition, the reduction of fungal burden is also a therapeutic target that can be addressed by antimycotic drugs, mainly itraconazole, with voriconazole or posaconazole used as alternatives in case of adverse reactions. In terms of clinical outcomes, ABPA treatment is aimed at obtaining symptom control, prevention of ABPA relapses and asthma exacerbations, and prevention of irreversible lung damage, while minimizing adverse drug effects. Current guidelines place systemic steroids (0.5 mg·kg^−1^·day^−1^ for 2–4 weeks of oral prednisolone equivalent, tapered and completed over 4 months) as the first‐line agents in ABPA acute phase and relapses, but itraconazole (400 mg·day^−1^, in two divided doses for 4 months) should also be considered, especially in the case of recurrent exacerbations [8]. One randomized trial showed faster and slightly higher response rates in glucocorticoid‐treated patients, however, at the price of increased treatment‐related side effects when compared to itraconazole [95]. Thus, in patients at risk for glucocorticoid toxicity, antifungal therapy is the preferred first‐line option. A combination of the two drugs should be considered in patients with frequent ABPA exacerbations [8].

Unfortunately, between 10% and 25% of ABPA patients develop a treatment dependence, needing multiple trials of systemic steroids and/or continuous antifungal therapy, potentially leading to increased risk of side effects, and no evidence of long‐term (years) efficacy and safety, and to the possibility of azole‐resistant Aspergillus strains selection [96]. Systemic corticosteroids are well known for their unfavorable safety profile, especially after long‐term/recurrent and/or high‐dose treatment regimens which are required for ABPA treatment, as mentioned above. In addition, the cumulative dose effect, even in the case of low daily doses, might result in a steroid‐related burden that is comparable to a higher exposure [97]. The most common effects include osteoporosis, eye conditions (cataracts and glaucoma), neuropsychiatric conditions, increased susceptibility to infections, and growth impairment in children. Steroids also increase fasting glucose, weight, blood pressure, and overall cardiovascular risk [98]. Azoles as well are burdened by significant toxicity mainly related to the liver, peripheral nervous system, and hormone dysregulations, especially in the case of a medium‐long‐term use [99]. Thus, the unfavorable security profile of first‐line therapies limits their prolonged use and provides the rationale for new therapeutic options.

The inflammatory background shared by asthma and ABPA, which commonly coexist, amplifying the overall disease burden, and the significant efficacy of anti‐T2 biologic agents in severe asthma patients in terms of symptoms improvement, steroid sparing effect, and exacerbation reduction, have provided the rationale for the off‐label use of the same options in ABPA.

Omalizumab, in light of its long standing on the market, collected the larger amount of evidence, including two small RCTs [100]. At the dose of 750 mg administered monthly, it showed a reduction in exacerbation and OCS use, associated with improved pulmonary function and asthma control [101, 102]. Currently, the advised dose is based on weight and total IgE levels, with a maximum dosage of 375 mg 2 times a month [8]. Of note, according to a recently published systematic review and meta‐analysis, no more than half of treated patients were able to completely withdraw the systemic OCS [101].

Anti IL‐5 agents mepolizumab and benralizumab consistently reduced exacerbations and OCS use in APBA patients in real‐life studies [103, 104], also demonstrating radiologic improvement with reduction in mucus plugging [104], particularly with benralizumab [75]. Notably, anti IL‐5 agents do not seem to reduce total IgE levels, even in patients with a good clinic‐radiological response. As the follow‐up duration in published studies is commonly no longer than 12 months, it is unclear whether persistently elevated IgE levels might predict, from a pathobiological perspective, a risk of relapse or poor control occurrence over time. However, in the short term, IgE should not be used to monitor drug efficacy in these patients [104]. Especially in the case of anti IL‐5 agents, besides the overall optimal efficacy profile, a certain degree of heterogeneity when looking at specific response domains, including lung function, exacerbation rate, and FeNO levels, should not be neglected [102, 104, 105]. The different disease phases and the degree of irreversible damage at the time of biologic treatment initiation might account for that.

Regarding dupilumab, multiple case reports and case series have shown a good response in terms of OCS sparing effect, reduction of exacerbations, mucus plugging improvement, even in patients not responding to other biologics [106, 107, 108, 109, 110]. However, the possible increase of blood eosinophil count that often occurs with dupilumab is still of some concern in the light of the commonly elevated baseline eosinophilia in ABPA patients. Results from a recently completed RCT of dupilumab in ABPA will hopefully provide further evidence (NCT04442269).

When looking at the data coming from asthma trials, the broader action of tezepelumab, an anti‐TSLP agent, has the potential to fully address the major drivers and clinical expressions of ABPA pathobiology, including mucus plugging [111]. Due to the recent approval of tezepelumab for severe asthma, evidence is still restricted to case reports [112, 113]. Of note, a post hoc analysis of pooled data from PATHWAY and NAVIGATOR trials demonstrated that in patients with uncontrolled asthma and fungal sensitization, tezepelumab reduced exacerbations versus placebo over 52 weeks, interestingly irrespective of blood eosinophil count at baseline and improved lung function and asthma symptoms from baseline to Week 52 [114].

None of the biological agents marketed for severe asthma is currently licensed for ABPA.

Generally speaking, when compared to the data on asthma patients, a less impressive effect characterizes biologic agents in ABPA patients [102], and when exacerbations occur even under biologic therapy, guidelines suggest the same management of a newly diagnosed ABPA [8].

The complex multiple‐driven immunological background underlying ABPA pathobiology might account for a lower biologic treatment response rate, but it should also be acknowledged the high heterogeneity of the published studies in terms of the timing of biologic therapy introduction in respect of ABPA disease stage (acute vs. relapsing vs. chronic) and in terms of clinical characteristics of treated populations (coexisting comorbidities, entity of lung irreversible impairment, disease duration). As a further point of weakness, when evaluating the baseline patient's profile and his response to biologic treatment, a clear differentiation between ABPA exacerbations vs. asthma exacerbations occurrence is not always clearly explored [102, 115].

However, the overall available evidence on biologic therapy in ABPA robustly supports its use, especially when severe asthma coexists. In fact, the last updated guidelines suggest monoclonal antibodies use in treatment‐dependent patients, that is, patients who need continuous therapies to maintain disease remission or who still relapse despite first‐line treatment [8]. So far, no specific recommendations are available on how to identify the best biologic treatment and on the best timing for its introduction.

Regarding the first point, taken together, the currently available evidence on biologic therapy options in ABPA patients, recently revised in the context of a systematic review and meta‐analysis [115], only partially supports the treatment selection process. Matching the specific drug‐related efficacy data and the patient's clinical/pathobiological profile might be helpful in identifying the better option. Under that perspective, OCS sparing effect, decreased exacerbation rate, improved clinical symptoms, and reduced radiographic abnormalities represent common outcomes which all the monoclonal antibodies have demonstrated to achieve in ABPA patients [37, 102]. Of note, some differences between specific biologic compounds can be highlighted in terms of: (i) lung function, significantly improved by omalizumab and dupilumab only;‐mucus plugging, better targeted by benralizumab and dupilumab; (ii) FeNO, more robustly impacted by mepolizumab; (iii) blood eosinophils, showing mepolizumab and benralizumab a greater decreasing effect; (iv) total IgE, which are more specifically addressed by dupilumab, mepolizumab, and omalizumab [102, 115]. Table 3 provides a graphical summary of the available evidence of different biologic compounds and specific efficacy outcomes. Tezepelumab was not included due to limited data relying on case reports only.

**TABLE 3:** Graphical summary of the current evidence on biologic compounds efficacy outcomes in ABPA patients (green cells indicate a demonstrated significant effect on the specific outcome).

Outcome	Biologic agent			
	*Benralizumab*	*Dupilumab*	*Mepolizumab*	*Omalizumab*
Systemic steroids				
Exacerbation rate				
Clinical symptoms				
Radiological abnormalities				
Total IgE				
Blood eosinophils				
FeNO				
Lung function				
Mucus plugging				

Abbreviation: FeNO, Fractional exhaled Nitric Oxide.

When considering the mechanistic perspective, the multiple pathways characterizing the interaction between AF and the airways epithelium discussed above support the relevance of biologic agents directly or indirectly interfering with the epithelial activation, namely dupilumab and tezepelumab. In fact, according to their mechanism, they have the potential to address both the IgE‐mediated hypersensitivity and the innate immunity‐dependent epithelial cytokines cascade [106, 111]. However, further data are needed to confirm the potential superiority of anti‐IL‐4/13 and anti‐TSLP agents. At the same time, eosinophilic inflammation is still considered a cardinal feature of the ABPA immunological background, interestingly irrespective of preceding asthma or atopic predisposition [70, 116]. This provides a strong rationale for the use of anti‐IL‐5 compounds as well. However, so far, a poor understanding of factors, including biomarkers and phenotype feature, predicting response to biologics or to a specific biologic therapy still hampers the positioning of those treatment options [102]. Interestingly, a recent study on 316 treatment‐naïve patients with ABPA identified allergic sensitization and HAM as determinants of difficult‐to‐treat ABPA [52], which might suggest an early introduction of biologic therapy in those cases and the selection of compounds selectively addressing mucus plug and IgE‐mediated cascade.

However, even more taking into account the limited data available so far, a careful patient profiling combined with each drug's evidence on specific outcomes should drive the treatment selection process and subsequent follow‐up. Proper dose regimens, timing of biologic initiation, possible combination of monoclonal antibodies to fully address the complex pathobiological ABPA background, tapering of biologic once remission has been achieved and maintained over time are just some of the open questions still unanswered.

### 5.1 Targeted Therapies in Children

Treatment goals in pediatric ABPA align with those in adults: reducing inflammation, controlling fungal burden, and preventing irreversible lung damage. Under that perspective, regular follow‐up is even more crucial than in asthma patients, with IgE monitoring every 3–6 months to detect relapses. Pulmonary function tests and imaging should be conducted periodically to assess disease progression [117].

Experts agreed that ABPA in children should be treated similarly to adults [8]. Oral corticosteroids remain the cornerstone of therapy, with a recommended starting dose of 0.5–1 mg/kg/day (max 40 mg) for 2–4 weeks, followed by a gradual taper over 3–6 months. However, in children, minimizing systemic steroid exposure is essential to prevent growth suppression and metabolic side effects [88].

Azole antifungals (itraconazole, voriconazole) play a critical adjunctive role by reducing *Aspergillus* colonization and steroid dependence; however, it is not currently recommended as first‐line treatment. Pediatric dosing should be carefully monitored for hepatotoxicity and drug interactions [118].

Data on biologics long‐term efficacy and safety in pediatric ABPA remain limited. Omalizumab (anti‐IgE) and mepolizumab (anti‐IL‐5) have shown promise in steroid‐sparing treatment for ABPA, particularly in children with concomitant severe eosinophilic asthma. However, their long‐term efficacy and safety in pediatric ABPA need further confirmation [88, 119].

## 6 Conclusion and Perspectives

The identification of pathobiological drivers underlying different conditions with potentially overlapping clinical manifestations remains challenging. It is the case of severe asthma, SAFS, and ABPA. In fact, available biomarkers, although accurate when related to each specific condition, do not always fully support a clear‐cut differential diagnosis. However, in the light of the diverse disease burden, potential trajectory, and available molecular tools allowing a truly early recognition, excluding a fungal‐driven immune response whether predominantly IgE‐mediated like in SAFS or multiple mechanisms like in ABPA, is mandatory in the baseline assessment of every patient affected by severe eosinophilic asthma. Furthermore, the same differential diagnosis should be applied when managing asthma exacerbations or loss of control during follow‐up, especially in the case of concomitant blood eosinophils increase.

The recent advances in terms of pathobiological mechanisms underlying asthma and ABPA, and in particular the central role of an epithelial‐driven impaired immune response especially in the case of ABPA, have revealed further connections between the two conditions and have highlighted new potential therapeutic targets. Under that perspective, some biologic options including dupilumab and perhaps even more tezepelumab might represent the way to address the key and early mechanisms of ABPA pathobiology. The opportunity for innovative targeted treatments, although needing further evidence, should further stimulate a precise endo‐phenotyping of severe asthma patients presenting some hallmarks of fungal‐related dis‐immunity to provide them the best standard of care for preventing disease evolution and achieving complete remission.

## Author Contributions

All authors contributed to the conception, structure, development, and review of the manuscript.

## Conflicts of Interest

The authors declare no conflicts of interest.

## Data Availability

Data sharing not applicable to this article as no datasets were generated or analyzed during the current study.
